# TPX2 expression is associated with poor survival in gastric cancer

**DOI:** 10.1186/s12957-016-1095-y

**Published:** 2017-01-09

**Authors:** Chiharu Tomii, Mikito Inokuchi, Yoko Takagi, Toshiaki Ishikawa, Sho Otsuki, Hiroyuki Uetake, Kazuyuki Kojima, Tatsuyuki Kawano

**Affiliations:** 1 Department of Gastrointestinal Surgery, Tokyo Medical and Dental University, 1-5-45, Yushima, Bunkyo-ku, Tokyo, 113-8519 Japan; 2 Department of Surgical Specialties, Tokyo Medical and Dental University, Tokyo, Japan; 3 Department of Minimally Invasive Surgery, Tokyo Medical and Dental University, Tokyo, Japan

**Keywords:** TPX2, Prognostic marker, Immunohistochemistry, Gastric cancer

## Abstract

**Background:**

Targeting protein for Xenopus kinesin-like protein 2 (TPX2) is a microtubule-associated protein required for microtubule formation in human cells. Several studies have demonstrated that TPX2 is overexpressed in multiple tumor types and promotes tumor growth and metastasis. However, there have been few reports regarding its role in gastric cancer. In this study, we evaluated TPX2 expression and investigated its correlations with gastric cancer clinicopathological features and prognosis.

**Methods:**

Tumor samples were obtained from 290 patients with gastric adenocarcinoma who had undergone gastrectomy. The expression of TPX2 protein was examined using immunohistochemical staining. *TPX2* messenger RNA (mRNA) levels were evaluated using real-time quantitative reverse transcription PCR in 19 of the gastric cancer tumors and adjacent normal tissues.

**Results:**

The mRNA levels of *TPX2* were significantly higher in gastric cancer tissues than in matched adjacent normal tissues (*p* = 0.004). In the immunohistochemical analysis, TPX2 overexpression was found in 123 (42.4%) of 290 patients. High TPX2 expression was positively associated with age, type of histology, depth of tumor, lymph node metastasis, stage, and remote metastasis or recurrence. High TPX2 expression was significantly associated with poorer disease-specific survival (*p* = 0.004) and relapse-free interval (*p* = 0.013).

**Conclusions:**

Our results indicated that high TPX2 expression was associated with tumor progression and poor survival in gastric cancer.

## Background

Although the mortality rate of gastric cancer has been declining for several decades, this disease remains one of the most common cancers [1]. Surgery is the main treatment for patients with localized disease, but even after macroscopic complete removal, many patients with advanced disease experience recurrence [2]. While combination chemotherapy regimens have been developed, overall survival ranges from 10 to 14 months in patients with unresectable or metastatic gastric cancer [3–5]. Therefore, the identification of prognostic biomarkers may contribute towards improving treatment strategies for gastric cancer patients.

Chromosomal instability and subsequent aneuploidy can promote tumor development [6]. Levels of targeting protein for Xenopus kinesin-like protein 2 (TPX2) were found to correlate with chromosomal instability [7]. High expression of TPX2 induces the amplification of centrosomes and leads to DNA polyploidy [8]. TPX2 is a microtubule-associated protein that is encoded by a gene located on human chromosome band 20q11.1. Its expression is tightly regulated by the cell cycle, and this protein is detected during the G1-S stage and disappears after the completion of mitosis. TPX2 is located in the nucleus during S-phase and G2 and at the spindle poles during mitosis. As a critical regulator of mitosis, TPX2 cooperates with Aurora-A kinase and Eg5 kinesin to control microtubule assembly and spindle stability. TPX2 is required for the formation of normal bipolar spindles and chromosome segregation [9, 10].

Overexpression of TPX2 has been reported in many types of tumors, including those from lung, hepatic, colon, pancreatic, salivary gland, and cervical cancers [11–18]. Moreover, TPX2 expression is a marker of worse tumor prognosis in several cancers [11–14]. These observations suggest that TPX2 plays a role in the oncogenesis of at least some malignancies. Nevertheless, there have been few studies that have reported investigation of the expression of TPX2 in gastric cancer. Therefore, in this study, we evaluated the expression of TPX2 in surgically resected specimens of gastric cancer and analyzed the association between TPX2 expression, clinicopathological factors, and survival.

## Methods

### Patients

Between January 2003 and December 2008, 290 patients underwent gastrectomy in the Department of Gastric Surgery of Tokyo Medical and Dental University. All patients were given sufficient explanation of the study, and written informed consent was obtained. This study was conducted in accordance with the Declaration of Helsinki and was approved by the Institutional Review Board of Tokyo Medical and Dental University.

Each tumor was classified according to the tumor-node-metastasis classification system recommended by the Union for International Cancer Control. Follow-up surveillance was performed every 3 to 6 months with computed tomography, abdominal ultrasonography, and tumor marker analysis. Patients with distant metastatic or recurrent disease received chemotherapy with S-1 (oral fluoropyrimidine consisting of tegafur, gimeracil, and oteracil potassium; Taiho Co, Tokyo, Japan) alone or combined chemotherapy. The median follow-up period was 62 months (range, 2–111 months). During follow-up, a total of 99 patients (34%) died as a result of their disease, 84 (29%) had recurrent disease, and 15 (5%) died of other causes.

### RNA extraction and cDNA synthesis

Immediately after surgery, small samples of gastric cancer tissue and adjacent normal tissue were collected, individually placed in RNA stabilization reagent (RNAlater; Qiagen, Valencia, CA, USA) and stored at −80 °C until analysis. Total RNA was extracted from each sample using the RNeasy Mini Kit (Qiagen) according to the manufacturer’s protocol. The concentration of total RNA was determined by absorption measurements at 260 and 280 nm using a UV spectrophotometer (Beckman Counter; Beckman Coulter, Brea, CA, USA). For complementary DNA (cDNA) synthesis, 10 μg of total RNA from each sample was reverse-transcribed into cDNA using the High Capacity cDNA Reverse Transcription Kit (Applied Biosystems, Foster City, CA, USA) according to the manufacturer’s protocol.

### Real-time quantitative reverse transcription PCR

Expression levels of *TPX2* and the gene for β-actin, which served as the endogenous control, were determined by real-time quantitative reverse transcription PCR (qRT-PCR) using the 7300 Real-Time PCR System (Applied Biosystems). TaqMan gene expression assays were purchased from Applied Biosystems (Hs00201616_m1). The PCR reaction was carried out using TaqMan Universal Master Mix II (Applied Biosystems) with 1 μl of cDNA in a 24-μl final reaction volume. Thermal cycling conditions were as follows: 2 min at 50 °C, 10 min at 95 °C, 40 cycles each of 15-s denaturation at 95 °C, and 1 min of annealing at 60 °C. The cDNA synthesized by the gastric cancer cell line MNK 45 was used for calibration. Each sample was run in duplicate for both the target gene and the endogenous control gene. The amount of *TPX2*, normalized to the endogenous control and relative to the calibration samples, was calculated by the comparative threshold cycle (Ct) method using Relative Quantification Study Software version 1.4 on the 7300 Sequence Detection System (Applied Biosystems).

### Immunohistochemical analysis of TPX2

Immunostaining was carried out with a peroxidase-labeled polymer conjugated to secondary antibodies (Histofine Simple Stain Max PO (MULTI), Nichirei Co., Tokyo, Japan). A monoclonal mouse antibody against TPX2 (sc-376812) was purchased from Santa Cruz Biotechnology, Inc. (Dallas, TX, USA.). All of the original hematoxylin and eosin-stained slides of the surgical specimens were reviewed if available, and representative paraffin blocks for each case were selected for immunohistochemistry. Four-micrometer-thick sections were cut from formalin-fixed, paraffin-embedded tissue blocks. After deparaffinization and rehydration, antigen retrieval treatment was conducted for 25 min in 1 × TE (1 × Tris-EDTA, pH 8.0) at 98 °C in a microwave processor (MI-77; AZUMAYA, Tokyo, Japan). Endogenous peroxidase activity was quenched by a 15-min incubation in a solution of 3% hydrogen peroxide in 100% methanol. After washing with phosphate-buffered saline (PBS), the slides were incubated with the primary monoclonal antibody against TPX2 (dilution 1:50) for 30 min at room temperature and then overnight (16 h) at 4 °C. The slides were washed three times with 0.1% Tween 20/PBS and were then incubated with Simple Stain Max PO (MULTI) for 30 min at room temperature. After three additional washes, 3,3’-diaminobenzidine tetrahydrochloride solution (Histofine Simple Stain DAB Solution; Nichirei Co., Tokyo, Japan) was applied to the slides, which were then counterstained with Mayer’s hematoxylin (Wako, Tokyo, Japan). Gastric cancer tissue from participants in this study that were strongly positive for TPX2 served as positive controls.

### Evaluation of immunohistochemistry for TPX2

In the tissue samples stained for TPX2, slides were considered positively stained when tumor cell nuclei were stained a darker brown than the background level of staining, regardless of intensity. TPX2 staining was graded as high (≥5% of tumor cells stained) or low (<5% of tumor cells stained). We stained representative cross-sectional slices of tumor tissue and counted at least five fields within most stained sections. The stained slides were evaluated by two independent observers (CT and YT) who were blinded to each patient’s prognosis. Any disagreements between the two observers were resolved by reassessment and consensus.

### Statistical analysis

The messenger RNA (mRNA) expression of *TPX2* in gastric cancer tissues and matched adjacent normal tissues was compared using paired *t* tests. The *χ* ^2^ test and the Mann-Whitney *U* test were used to analyze the association between TPX2 protein expression and clinicopathological factors. Kaplan-Meier curves were compared using a log-rank test for univariate analysis of disease-specific survival (DSS) and relapse-free interval (RFI). For the RFI analysis, 25 stage IV patients were excluded. In all analyses, IBM SPSS Statistics Software version 22 (IBM, Armonk, NY, USA) was used, and *p* values <0.05 were considered significant.

## Results

### mRNA expression of *TPX2*

**Fig. 1:**
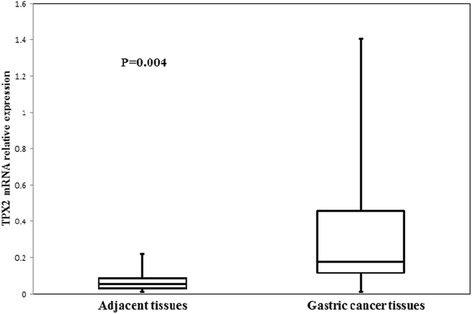
Targeting protein for Xenopus kinesin-like protein 2 (*TPX2*) mRNA expression in gastric cancerous and adjacent normal tissues. The median *TPX2* mRNA level was significantly higher in gastric cancer than in the adjacent normal tissue in 19 patients (*p* = 0.004)

Initially, we assayed *TPX2* mRNA levels in a retrospective cohort of 19 gastric cancer tissues and matched adjacent normal tissues using qRT-PCR. In this cohort, the median *TPX2* mRNA level was significantly higher in gastric cancer tissues than in matched adjacent normal tissues (*p* = 0.004; Fig. 1).

### Immunohistochemistry for TPX2 expression

**Fig. 2:**
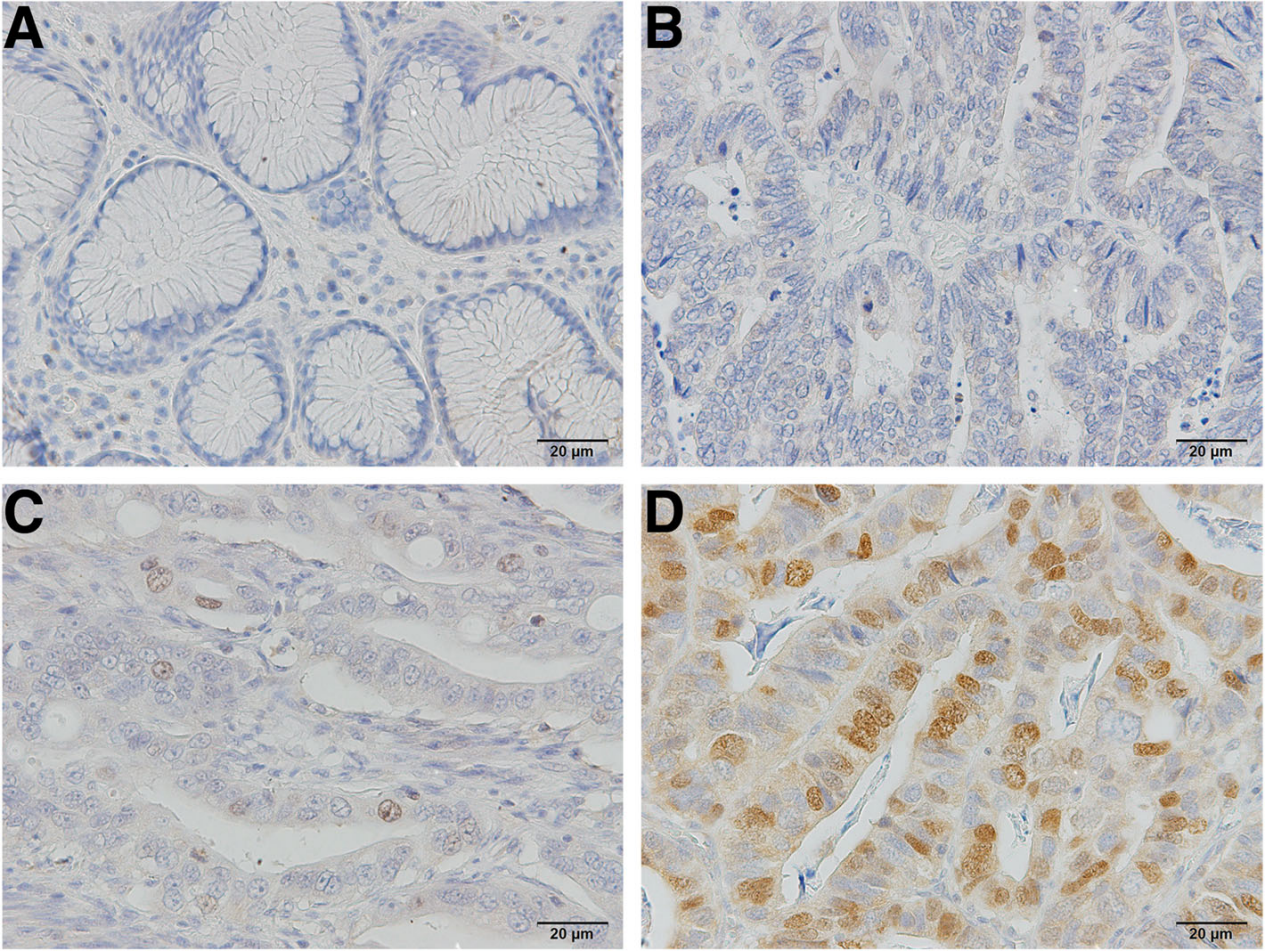
Representative photomicrographs of immunostaining for targeting protein for Xenopus kinesin-like protein 2 (TPX2), demonstrating **a** negative staining in normal gastric epithelium, **b** negative staining in primary gastric carcinoma, **c** positive staining graded as low (<5% of tumor cells stained), and **d** positive staining graded as high (≥5% of tumor cells stained). TPX2 protein was mainly localized within the nuclei of tumor cells. Images were captured at ×200 magnification

Expression of the TPX2 protein was assessed by immunohistochemical staining of gastric cancer tissue samples from 290 patients. Of these samples, 123 (42.4%) were classified as having high TPX2 expression (≥5% of tumor cells stained). TPX2 expression was mainly observed in the nuclei of tumor cells, with no expression observed in the nuclei of cells in normal tissue (Fig. 2). Weak expression was observed in the cytoplasm of some tumor cells.

### Relationship between TPX2 and clinicopathological factors

High expression of TPX2 was significantly associated with the patient’s age (<65 vs. ≥65, *p* < 0.001), type of histology (differentiated vs. undifferentiated, *p* = 0.002), depth of tumor (T1 vs. T2 vs. T3 vs. T4, <0.001), lymph node metastasis (N0 vs. N1 vs. N2 vs. N3, *p* < 0.001), stage (I vs. II vs. III vs. IV, *p* < 0.001), and remote metastasis or recurrence (presence vs. absence, *p* = 0.02; Table 1).

**Table 1:** Relationship between expression of TPX2 and patient clinicopathological factors

		TPX2 expression		
	*n*	Low (*n* = 167)	High (*n* = 123)	*p* value
Gender				
Male	219	127	92	
Female	71	40	31	0.807
Age				
<65	129	88	41	
≥65	161	79	82	0.001
Pathological type				
Undifferentiated	158	104	54	
Differentiated	132	63	69	0.002
Main location				
Middle or lower	233	135	98	
Upper	57	32	25	0.805
Depth of invasion				
T1	120	83	37	
T2	37	22	15	
T3	49	24	25	
T4	84	38	46	<0.001
LN metastasis				
N0	149	104	45	
N1	44	26	18	
N2	39	12	27	
N3	58	25	33	<0.001
Stage				
I	139	98	41	
II	51	26	25	
III	75	30	45	
IV	25	13	12	<0.001
Distant metastasis or recurrence				
Negative	205	127	78	
Positive	85	40	45	0.020

*TPX2* targeting protein for Xenopus kinesin-like protein 2, *LN* lymph node

### Relationship between TPX2 and survival

**Fig. 3:**
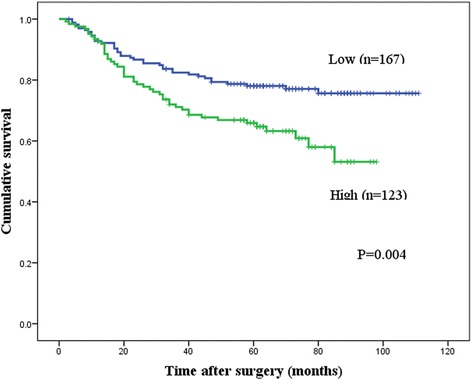
Kaplan-Meier curves for the disease-specific survival (DSS) of patients with targeting protein for Xenopus kinesin-like protein 2 (TPX2) expression. Patients with high TPX2 protein expression (*n* = 123) had significantly poorer DSS than those with low TPX2 expression (*n* = 167; *p* = 0.004)

**Fig. 4:**
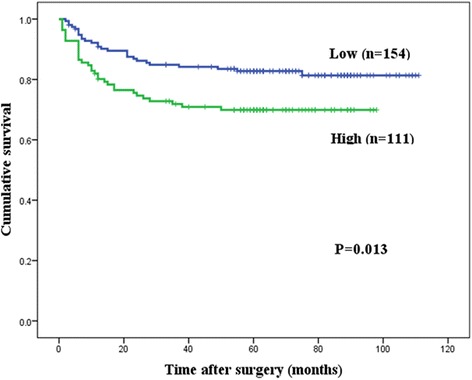
Kaplan-Meier curves for the relapse-free interval (RFI) of patients with targeting protein for Xenopus kinesin-like protein 2 (TPX2) expression. Patients with high TPX2 protein expression (*n* = 123) had significantly poorer RFI than those with low TPX2 expression (*n* = 167; *p* = 0.013)

High expression of TPX2 was associated with poorer DSS (*p* = 0.004) and RFI (*p* = 0.013) by univariate analysis. In patients with positive expression of TPX2, the 5-year DSS was 65.9% and the RFI was 69.9%; in patients with negative expression of TPX2, these values were 78.1 and 82.8%, respectively (Figs. 3 and 4).

**Fig. 5:**
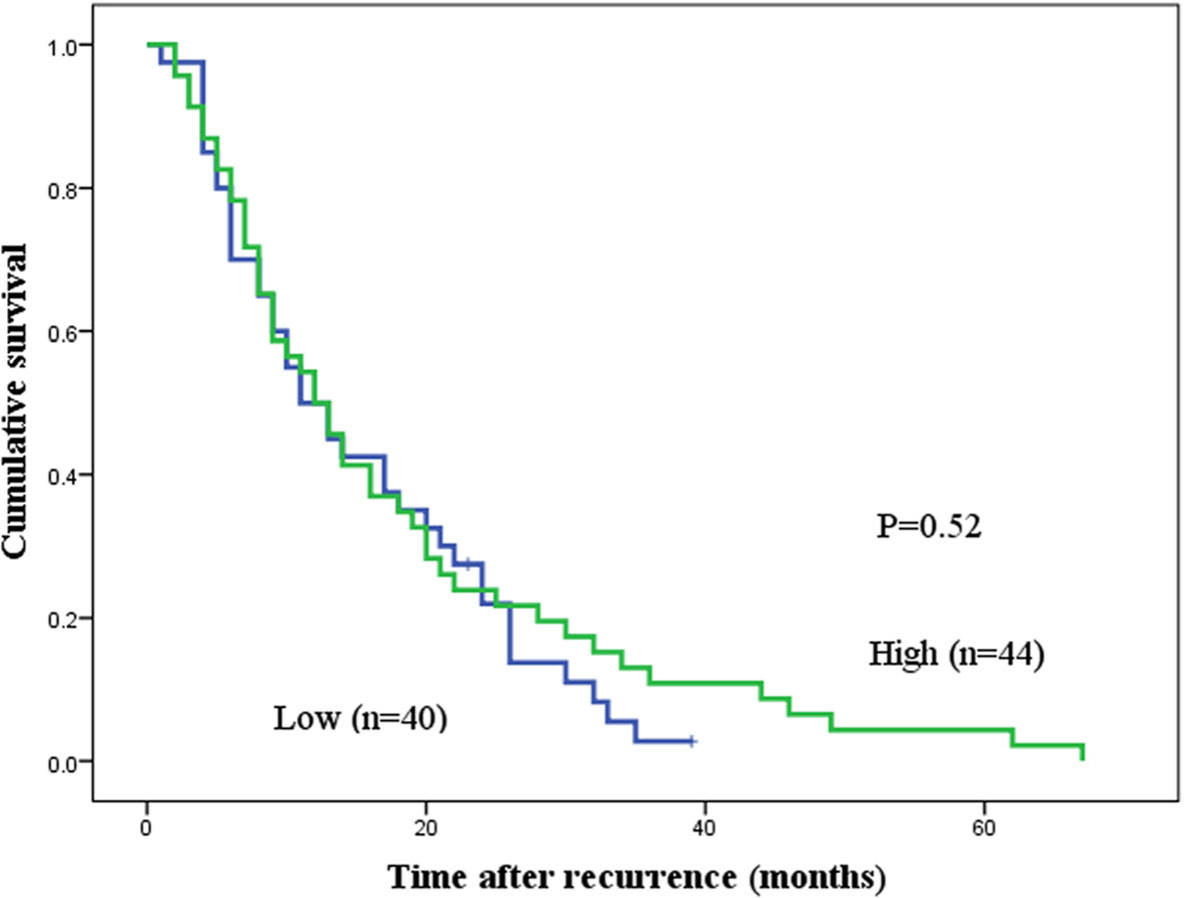
Kaplan-Meier curves for disease-specific survival (DSS) after recurrence of patients with low or high levels of targeting protein for Xenopus kinesin-like protein 2 (TPX2) expression. The log-rank test showed no significant difference between DSS and TPX2 expression in patients after recurrence (*p* = 0.52)

After adjustment for significant prognostic factors (age, histopathology, tumor location, depth of invasion, lymph node involvement), the multivariate analysis of clinicopathological features affecting DSS indicated that independent prognostic factors were depth of tumor invasion (hazard ratio (HR) 7.7, 95% CI 2.7 to 21.6, *p* < 0.001) and lymph node metastasis (HR 6.4, 95% CI 3.1 to 13.2, *p* < 0.001; Table 2). After adjustment for the same significant prognostic factors, multivariate analysis of clinicopathological features affecting RFS indicated that independent prognostic factors were tumor main location (HR 1.9, 95% CI 1.1 to 3.3, *p* = 0.02), depth of tumor invasion (HR 6.0, 95% CI 2.1 to 17.1, *p* = 0.001), and lymph node metastasis (HR 6.7, 95% CI 3.0 to 15.3, *p* < 0.001; Table 3). High TPX2 expression was not an independent prognostic factor for either DSS or RFS. Comparison of Kaplan-Meier curves of the DSS of patients with low and high TPX2 expression after recurrence by using the log-rank test showed no significant difference between the DSS of these two groups (*p* = 0.52; Fig. 5).

**Table 2:** Prognostic factors for disease-specific survival (DSS) in univariable and multivariable Cox proportional hazards regression models

	Univariate (log-rank)		Multivariate		
	5-year DSS (%)	*p* value	HR	95% CI	*p* value
Gender					
Male	72.3				
Female	74.6	0.891			
Age					
<65	78.8		1		
≥65	68.1	0.025	1.42	0.90 to 2.26	0.136
Pathological type					
Differentiated	82.0		1		
Undifferentiated	65.4	0.004	1.32	0.82 to 2.12	0.253
Main location					
Middle or lower	76.1		1		
Upper	59.7	0.026	1.52	0.94 to 2.47	0.089
Depth of invasion					
T1	97.4		1		
T2-T4	56.0	<0.001	7.66	2.71 to 21.6	<0.001
LN metastasis					
Negative	94.5		1		
Positive	50.0	<0.001	6.40	3.10 to 13.2	<0.001
TPX2					
Low	78.1		1		
High	65.9	0.004	1.05	0.66 to 2.26	0.840

*DSS* disease-free survival, *TPX2* targeting protein for Xenopus kinesin-like protein 2, *LN* lymph node

**Table 3:** Prognostic factors for relapse-free interval (RFI) in univariable and multivariable Cox proportional hazards regression models

	Univariate (log-rank)		Multivariate		
	5-year RFI (%)	*p* value	HR	95% CI	*p* value
Gender					
Male	76.6				
Female	79.7	0.791			
Age					
<65	79.4				
≥65	75.7	0.323			
Pathological type					
Differentiated	85.2		1		
Undifferentiated	70.5	0.004	1.46	0.82 to 2.57	0.196
Main location					
Middle or lower	81.4		1		
Upper	60.9	0.003	1.93	1.12 to 3.31	0.017
Depth of invasion					
T1	96.6		1		
T2-T4	61.7	<0.001	6.01	2.11 to 17.1	0.001
LN metastasis					
Negative	95.2		1		
Positive	54.7	<0.001	6.72	2.95 to 15.3	<0.001
TPX2					
Low	82.8		1		
High	69.9	0.013	1.16	0.68 to 1.96	0.590

*RFI* relapse-free interval, *TPX2* targeting protein for Xenopus kinesin-like protein 2, *LN* lymph node

## Discussion

In this study, we used a large set of surgically resected gastric cancer tissue samples to assess the expression of TPX2 at the protein level. Our results indicated that high expression of TPX2 may play a critical role in tumor progression, metastasis, and survival in gastric cancer.

TPX2 expression in samples of human gastric cancer has been investigated in one previous study [19]. Although that study differed from ours in having a smaller sample size and different evaluation of the immunohistochemical results, their findings were similar to those of our study. Those authors reported that overexpression of TPX2 was related to advanced stages and poor survival. Moreover, they further reported that *TPX2*-siRNA suppressed tumor cell epithelial-mesenchymal transition in gastric cancer cell lines [19].

A number of studies have examined the relationship between TPX2 and human malignancies. TPX2 is highly expressed at both the mRNA and protein level in many types of cancers, including lung, hepatic, colon, pancreatic, salivary gland, and cervical cancers [11–18]. Using immunostaining, multiple studies reported that TPX2 is mainly localized within the nuclei of cancer cells [11–18]. TPX2 expression detected by immunohistochemical analysis was associated with depth of tumor, lymph node metastasis, and remote metastasis in colon cancer [13]. TPX2 expression analyzed by immunohistochemistry was associated with poor patient survival in many types of cancer [11–14]. TPX2 is an independent prognostic indicator for poor patient survival in hepatic cancer [14]. In colon cancer, TPX2 is strongly associated with the progression of colorectal adenoma to carcinoma [16].

TPX2 has been identified as a microtubule-associated protein that is required for bipolar spindle assembly. TPX2 recruits Aurora-A kinase to microtubules and drives the activation of this kinase during mitosis. TPX2-Aurora-A binding is promoted by an active Ran-GTP signaling pathway [9, 10]. The critical role of TPX2 in mitosis is supported by several studies. In HeLa cells, treatment with *TPX2*-siRNA caused defects in microtubule organization during mitosis, with the consequence that microtubule asters could not be formed and the spindle could not be assembled [8]. In mouse embryonic fibroblasts, *TPX2*-siRNA-treated embryos arrested at the morula stage with defective mitotic spindles [20]. Moreover, *TPX2*-siRNA treatment resulted in the generation of tetraploid and aneuploid cells [20]. These preclinical studies suggest that TPX2 is required for the formation of normal bipolar spindles and normal cell division.

Many studies suggest that decreasing TPX2 levels may be a beneficial approach for cancer treatment. For instance, *TPX2*-siRNA decreased the viability and proliferation capacity of colon, cervical, and hepatic cancer cell lines [13, 14, 18]. Similarly, *TPX2*-siRNA induced cell apoptosis in hepatic cancer cell lines [12]. Moreover, injection of *TPX2*-siRNA significantly reduced the growth and weight of already developed xenograft tumors in nude mice [13–15, 21, 22]. TPX2 plays an important role in the mechanism of paclitaxel’s activity against cancers [23]. Warner et al. suggested that the exposure of pancreatic cancer cells to *TPX2*-siRNA plus paclitaxel results in a synergistic decrease in cell viability [15]. These observations suggest that targeted inactivation of TPX2 may have therapeutic benefits. However, TPX2 inhibitors have not been developed, and most current therapies are designed to inhibit its partner, Aurora-A, using small-molecule kinase inhibitors [24].

Several limitations of this study should be acknowledged. First, gene expression of *TPX2* was assessed in only 19 tissues. However, in the Oncomine database, high levels of *TPX2* mRNA and copy number gain of the *TPX2* gene are shown in some datasets of human gastric cancers [25]. Further studies are required to assess more patient samples for *TPX2* gene expression and to further explore its clinical significance. Second, in our evaluation of immunohistochemistry slides, we set the cutoff point for a positive immunoreactive score at 5% of the cells. However, studies of other types of cancers have set the immunoreactivity cutoff point at 10% [11, 17]. In gastric cancer, there is no consensus for a cutoff point; furthermore, we observed that few cases displayed staining in more than 10% of the cells.

## Conclusions

We found that high TPX2 expression was associated with tumor progression and poor survival in gastric cancer patients. TPX2 may thus play an important role in tumor progression in gastric cancer.

## Abbreviations

TPX2: Targeting protein for Xenopus kinesin-like protein 2
PBS: Phosphate-buffered saline
DSS: Disease-free survival
RFI: Relapse-free interval
HR: Hazard ratio
